# Seizure burden and neurodevelopmental outcome in neonates with hypoxic–ischemic encephalopathy

**DOI:** 10.1111/dmcn.13215

**Published:** 2016-09-06

**Authors:** Liudmila Kharoshankaya, Nathan J Stevenson, Vicki Livingstone, Deirdre M Murray, Brendan P Murphy, Caroline E Ahearne, Geraldine B Boylan

**Affiliations:** ^1^ Irish Centre for Fetal and Neonatal Translational Research (INFANT)CorkIreland; ^2^ Department of Paediatrics and Child HealthUniversity College CorkCorkIreland; ^3^ Department of NeonatologyCork University Maternity HospitalCorkIreland

## Abstract

**Aim:**

To examine the relationship between electrographic seizures and long‐term outcome in neonates with hypoxic–ischemic encephalopathy (HIE).

**Method:**

Full‐term neonates with HIE born in Cork University Maternity Hospital from 2003 to 2006 (pre‐hypothermia era) and 2009 to 2012 (hypothermia era) were included in this observational study. All had early continuous electroencephalography monitoring. All electrographic seizures were annotated. The total seizure burden and hourly seizure burden were calculated. Outcome (normal/abnormal) was assessed at 24 to 48 months in surviving neonates using either the Bayley Scales of Infant and Toddler Development, Third Edition or the Griffiths Mental Development Scales; a diagnosis of cerebral palsy or epilepsy was also considered an abnormal outcome.

**Results:**

Continuous electroencephalography was recorded for a median of 57.1 hours (interquartile range 33.5–80.5h) in 47 neonates (31 males, 16 females); 29 out of 47 (62%) had electrographic seizures and 25 out of 47 (53%) had an abnormal outcome. The presence of seizures per se was not associated with abnormal outcome (*p*=0.126); however, the odds of an abnormal outcome increased over ninefold (odds ratio [OR] 9.56; 95% confidence interval [95% CI] 2.43–37.67) if a neonate had a total seizure burden of more than 40 minutes (*p*=0.001), and eightfold (OR: 8.00; 95% CI: 2.06–31.07) if a neonate had a maximum hourly seizure burden of more than 13 minutes per hour (*p*=0.003). Controlling for electrographic HIE grade or treatment with hypothermia did not change the direction of the relationship between seizure burden and outcome.

**Interpretation:**

In HIE, a high electrographic seizure burden is significantly associated with abnormal outcome, independent of HIE severity or treatment with hypothermia.

## What this paper adds

- In hypoxic–ischemic encephalopathy (HIE), high electrographic seizure burden is significantly associated with abnormal outcome.
- The association is independent of HIE severity or treatment with hypothermia.
- The presence of seizures per se is not associated with abnormal outcome.

This article is commented on by Kirkpatrick and Jollands on page 1206 of this issue.

**Abbreviations**
AED: Antiepileptic drug
aEEG: Amplitude‐integrated electroencephalography
Bayley‐III: Bayley Scales of Infant and Toddler Development, Third Edition
cEEG: Continuous electroencephalography
HIE: Hypoxic–ischemic encephalopathy
IQR: Interquartile range
MSB: Maximum seizure burden
ROC curve: Receiver operator characteristic curve
TSB: Total seizure burden

Hypoxic–ischemic encephalopathy (HIE) is a common cause of seizures in term neonates. In HIE, seizures signal the secondary injury phase and may further aggravate brain damage.^1^, ^2^, ^3^ Numerous studies in asphyxiated neonates before the advent of therapeutic hypothermia have shown a relationship between the presence of clinical and electrographic seizures, especially status epilepticus, and an abnormal outcome.^2^, ^4^, ^5^, ^6^, ^7^ Most of these studies used clinical assessment or amplitude‐integrated electroencephalography (aEEG) for seizure detection and quantification. In addition, many assessed only short‐term outcome, analysed cohorts of mixed aetiology, or did not control for confounding variables. Owing to the difficulty of separating the severity of the underlying injury from the effect of seizures themselves, debate persists about the impact of seizures on brain injury and outcome.^8^, ^9^

The advent of therapeutic hypothermia has inspired researchers to re‐evaluate the relationship between seizures and outcome, as therapeutic hypothermia has been shown to reduce total seizure burden (TSB)^10^ and improve outcomes in HIE.^11^ Several studies in neonates with HIE undergoing hypothermia have shown that electrographic seizures and high seizure burden are associated with more severe brain injury on magnetic resonance imaging (MRI).^12^, ^13^, ^14^ In a recent randomized anti‐seizure treatment trial in neonates with HIE, most of whom were treated with hypothermia, a higher TSB on continuous electroencephalography (cEEG) was associated with lower scores on the Bayley Scales of Infant and Toddler Development, Third Edition (Bayley‐III) at 18 to 24 months of age. The results were not controlled for HIE severity or therapeutic hypothermia, and hourly seizure burdens were not quantified.^14^

We used cEEG to examine the relationship between neonatal seizures, seizure burden, and developmental outcome at 24 to 48 months of age in neonates with moderate and severe HIE treated and not treated with hypothermia.

## Method

### Patients

This study included neonates at more than and including 37 weeks’ gestation, born in Cork University Maternity Hospital in 2003 to 2006 (pre‐hypothermia era) and in 2009 to 2012 (hypothermia era), who fulfilled the criteria of perinatal asphyxia and who had clinical signs of evolving HIE (mild, moderate, and severe).^4^ We excluded neonates with evidence of perinatal infection, metabolic or congenital abnormalities. This study was a retrospective analysis of two cohorts of neonates prospectively recruited to cEEG studies of neonates at high risk of seizures.^4^, ^15^ The modified Sarnat score (Levene) for HIE was performed in all neonates at 24 hours after birth during recruitment of both studies.

### cEEG recordings and HIE grading

All neonates underwent video‐cEEG monitoring (Nicolet One ICU Monitor, Neuro‐Care; Carefusion, Middleton, WI, USA) with active electrodes applied at F3, F4, C3, C4, T3, T4, O1, O2, and Cz (international 10–20 system of electrode placement modified for neonates), with single‐channel electrocardiography and respiration monitoring. The aEEG signal was displayed simultaneously. cEEG recordings were reviewed retrospectively in their entirety by an experienced neonatal neurophysiologist (GBB).

The overall severity of HIE was determined electrographically at 24 hours after birth on the basis of cEEG background characteristics, presence of sleep–wake cycle, and electrographic seizures (Table 1).^4^ The electrographic grading of HIE severity was selected as a less subjective method over clinical HIE grading.^11^ Neonates with mildly or moderately abnormal cEEG background patterns were considered to have mild or moderate electrographic HIE grades respectively; neonates with majorly abnormal or inactive patterns were combined into one group designated as a severe electrographic HIE grade. If the cEEG was graded as mildly abnormal at 24 hours after birth but electrographic seizures were present at any time, the cEEG recording received a moderate electrographic grade of HIE. In our analysis, we excluded all neonates with a mild electrographic HIE grade at 24 hours after birth. We also excluded cases where monitoring was initiated beyond 24 hours of birth or if monitoring was less than 24 hours in total duration.

**Table 1:** Clinical characteristics of neonates with normal and abnormal outcome included in the study (*n*=47)

	Normal outcome (*n*=22)^a^		Abnormal outcome (*n*=25)^a^		*p* b
	*n*	%	*n*	%	
Sex					
Male	13	59.1	18	72.0	0.376
Female	9	40.9	7	28.0	
Clinical Sarnat HIE grade					
Mild	5	22.7	2	8.0	0.018
Moderate	14	63.6	10	40.0	
Severe	3	13.6	13	52.0	
cEEG‐HIE grade					
Moderate	18	81.8	6	24.0	<0.001
Severe	4	18.2	19	76.0	
Therapeutic hypothermia status					
Therapeutic hypothermia	13	59.1	7	28.0	0.042
Non‐therapeutic hypothermia	9	40.9	18	72.0	
Number of AEDs^c^^,^ d					
<3	21	95.5	9	37.5	<0.001
≥3	1	4.5	15	62.5	
	Median	IQR	Median	IQR	*p* **e**
Gestational age (wk)	40.7	38.9–41.1	40.5	39.7–41.6	0.536
Birthweight (g)	3500	3163–3910	3450	3045–3815	0.991
Apgar at 5min^c^	5	4–6	5	2–7	0.807

^a^Unless otherwise stated. ^b^From Fisher's exact test. ^c^ *n*=24 for abnormal outcome group. ^d^Thirty‐one neonates in the cohort received AED. ^e^From Mann–Whitney *U* test. cEEG, continuous electroencephalography; HIE, hypoxic–ischemic encephalopathy; AED, antiepileptic drug; IQR, interquartile range.

### Seizure analysis and quantification

All seizures in each entire cEEG recording were annotated visually. Electrographic seizure was defined as a sudden repetitive, stereotyped discharge of minimum 10 seconds’ duration on one or more EEG channels with evolving frequency, amplitude, and morphology.^16^ The annotations were used to calculate TSB and hourly seizure burden for each neonate. Following this analysis, two summary measures of seizures over different time scales were defined: TSB and maximum seizure burden (MSB). The TSB summarizes the duration of all seizures on the EEG recording for each patient individually and is measured in minutes. The MSB is defined as the maximum hourly seizure burden within cEEG recording and is measured in minutes per hour.

Seizure period was also calculated as the time from the onset of the first electrographic seizure to the offset of the last electrographic seizure.

Administration of antiepileptic drugs (AEDs) was recorded and included phenobarbitone as first‐line medication, followed by phenytoin and midazolam as second‐ and a third‐line medications respectively. Lidocaine and levetiracetam were also used in refractory cases.

The antiepileptic protocols and general treatment, besides therapeutic hypothermia, did not differ between the two periods (pre‐hypothermia and hypothermia eras). All clinical seizures in the cohort were treated. Neonatologists used the aEEG display to aid seizure treatment decisions when necessary. Immediate cEEG interpretation was not always available, but, in the case of suspected seizures on aEEG or clinical seizures, the cEEG was interpreted by encephalographers as soon as possible and all seizures detected were treated.

### Outcome

Neurodevelopmental outcome was assessed using either Bayley‐III (by CEA) or the Griffiths Mental Development Scales (by DMM) at the age of 24 to 48 months. The assessors were blind to the neonatal clinical course and cEEG findings. Griffiths assessment was performed in the cohort recruited between 2003 and 2006 (Murray et al.^4^) and in neonates recruited in 2009 to 2012 who were older than 42 months at the time of the assessment.

The outcome was graded as binary (normal/abnormal). We considered outcome as abnormal in the case of death, dyskinetic or spastic quadriplegic CP, epilepsy diagnosed by a paediatric neurologist, a Griffiths general quotient (GQ) <87, or more than two SDs below the mean in any individual subquotient; and a Bayley‐III composite score <85 in all three subscales or <70 in any individual subscale.

### Statistical analysis

Statistical analysis was performed using IBM SPSS Statistics (version 22.0; IBM Corp., Armonk, NY, USA). Continuous variables were described using median (interquartile range [IQR]) and categorical data using frequency (%). For comparisons between groups, the Mann–Whitney *U* test was used for continuous variables and Fisher's exact test for categorical variables. The association between TSB, MSB, and outcome was assessed using receiver operator characteristic (ROC) curve analysis. Optimal cutoff points of TSB and MSB were chosen as the point on the ROC curve closest to the upper left corner (where sensitivity=1 and specificity=1), often known as the (0,1) criterion. Diagnostic accuracy was assessed using positive predictive value, negative predictive value, and area under the ROC curve and their corresponding 95% confidence intervals (CIs). Univariable and multivariable logistic regression analyses estimated unadjusted and adjusted odds ratios (OR) and 95% CIs of an abnormal outcome. All tests were two‐sided and a *p*‐value <0.05 was considered to be statistically significant.

### Ethical approval

The clinical Research Ethics Committee of the Cork Teaching Hospitals approved this study. Written informed consent was obtained from the parents of all children studied. Patient data were anonymized before analysis.

## Results

During the recruitment periods, cEEG was recorded in 100 neonates with HIE. The electrographic HIE grade at 24 hours was mild in 40, moderate in 32, and severe in 28. Of these, 53 neonates were excluded: 40 neonates had a mild cEEG grade; in six neonates the cEEG recording started beyond 24 hours of birth or was less than 24 hours in duration; and seven neonates were lost to follow‐up. This resulted in a study cohort of 47 neonates: 24 with moderate HIE (15 non‐therapeutic hypothermia and 9 therapeutic hypothermia) and 23 with severe HIE (12 non‐therapeutic hypothermia and 11 therapeutic hypothermia). Clinical characteristics are presented in Table 1.

cEEG monitoring was started at a median age of 6 hours after birth (IQR 3.5–11.0) and continued for a median of 57.1 hours (IQR 33.5–80.5). Twenty‐nine out of 47 neonates had electrographic seizures. Seizure characteristics in relation to therapeutic hypothermia subgroups are shown in Table 2.

**Table 2:** Seizure characteristics in neonates treated with and without therapeutic hypothermia in the cohort (*n*=47)

	Non‐therapeutic hypothermia (*n*=27)^a^		Therapeutic hypothermia (*n*=20)^a^		*p* b
	Median	IQR^a^	Median	IQR^a^	
Start of recording (h after birth)	10.2	4.9–14.0	3.8	3.3–6.0	0.002
Duration of EEG recording (h)	37.2	25.7–55.6	82.0	76.5–92.0	<0.001
Seizure frequency: *n* (%)	17	63.0	12	60.0	1^c^
Seizure frequency in moderate HIE: *n* (%)	8/15	53.3	3/9	33.3	0.423^c^
Seizure frequency in severe HIE: *n* (%)	9/12	75.0	9/11	81.8	1^c^
Seizure onset (h after birth)^d^	15.3	11.6–21.3	13.7	11.7–18.4	0.711
Time of MSB (h after birth)^d^	19.6	17.3–29.4	17.3	15.0–21.8	0.211
Seizure period (h)^d^	33.2	13.0–46.0	14.0	6.3–37.0	0.283
TSB (min)^d^	183.2	45.1–311.3	55.8	22.4–145.2	0.097
TSB in moderate HIE (min)^d^	87.9	11.8–197.4	24.1	21.8–118.9^e^	0.497
TSB in severe HIE (min)^d^	206.1	144.4–510.1	67.2	28.5–176.1	0.019
MSB (min/h)^d^	25.1	12.6–47.0	18.5	9.6–33.3	0.419
MSB in moderate HIE (min/h)^d^	12.6	7.8–33.4	21.8	8.6–30.2^e^	1
MSB in severe HIE (min/h)^d^	45	22.7–50.8	15.2	10.1–38.5	0.094

^a^Unless otherwise stated. ^b^From Mann–Whitney *U* test unless otherwise stated. ^c^From Fisher's exact test. ^d^Seizure infants only. ^e^Minimum to maximum as *n*=3. IQR, interquartile range; EEG, encephalography; HIE, hypoxic–ischemic encephalopathy; MSB, maximum seizure burden TSB, total seizure burden.

In the cohort, 31 neonates received AEDs. Seven neonates were treated with AEDs for suspected clinical seizures before cEEG monitoring and/or during cEEG monitoring without further confirmation of electrographic seizures. Thirteen out of 31 neonates received AEDs before cEEG monitoring initiation (five in the therapeutic hypothermia subgroup [*n*=2 were seizure free] and eight in the non‐therapeutic hypothermia subgroup [*n*=2 were seizure free]). Four neonates with electrographic seizures did not receive AEDs.

An abnormal outcome was seen in 25 out of 47 neonates: seven infants died (five neonates with severe encephalopathy died during the early neonatal period, and two infants with severe spastic quadriplegia died at 4 months and 24 months of age after recurrent respiratory infections); five of those who died had electrographic seizures; 12 other patients survived with CP (10 seizure and two non‐seizure), and six further infants had abnormal Bayley‐III (*n*=2, one seizure and one non‐seizure) or Griffiths assessments (*n*=4, two with seizures and two without seizures).

Five out of 40 survivors developed epilepsy (all five were from the group with CP and had electrographic seizures). The severity of HIE grade at 24 hours after birth and the absence of treatment with therapeutic hypothermia were significantly associated with abnormal outcome at 24 to 48 months (*p*<0.001 and *p*=0.042 respectively; Table I).

### Assessment of seizures and outcome

The percentage of infants with an abnormal outcome was higher in the seizure group: 62% (18 out of 29) compared with 39% (7 out of 18) in the non‐seizure group; however, this difference did not reach statistical significance (*p*=0.126; Table 3).

**Table 3:** Logistic regression analysis showing relationships between seizures, high total seizure burden (TSB; >40min), high maximum seizure burden (MSB; >13min/h), and abnormal outcome (*n*=47)

	Unadjusted			Adjusted for cEEG‐HIE grade			Adjusted for therapeutic hypothermia		
	OR	95% CI	*p*	OR	95% CI	*p*	OR	95% CI	*p*
Seizure (*n*=29)	2.57	0.77–8.61	0.126	1.25	0.28–5.55	0.769	2.74	0.76–9.88	0.123
TSB >40min (*n*=21)	9.56	2.43–37.67	0.001	4.84	1.04–22.65	0.045	11.42	2.51–51.99	0.002
MSB >13min (*n*=20)	8.00	2.06–31.07	0.003	5.57	1.18–26.28	0.030	8.21	1.96–34.42	0.004

cEEG, multi‐channel electroencephalogram; HIE, hypoxic–ischemic encephalopathy; OR, odds ratio; 95% CI, 95% confidence interval.

### Assessment of seizure burden and outcome in the cohort

TSB was significantly higher in neonates with an abnormal outcome (median [IQR] 151.60 [0–225.85] min, *n*=25) than in neonates with a normal outcome (median [IQR] 2.35 [0–28.60] min, *n*=22, *p*=0.002). MSB was also significantly higher in the group with an abnormal outcome (median [IQR] 25.10 [0–45.60] min/h) than in the group with a normal outcome (median [IQR] 2.35 [0–11.95] min/h, *p*=0.002). Based on the ROC curve analysis, the optimal cut‐off point to predict abnormal outcome was 40 minutes for TSB and 13 minutes per hour for MSB.

A TSB of more than 40 minutes (high TSB) (*n*=21) and an MSB of more than 13 minutes per hour (high MSB) (*n*=20) significantly increased the risk of the abnormal outcome (*p*=0.001 and *p*=0.003 respectively). This increase was independent of the electrographic grade of HIE at 24 hours after birth or the application of therapeutic hypothermia (Table 3).

The area under the ROC curve was 0.75 (95% CI 0.60–0.86) for a high TSB and 0.73 (95% CI 0.57–0.84) for a high MSB. The positive predictive value of a high TSB for abnormal outcome was 81%, while the negative predictive value was 69%. A high MSB had a positive predictive value of 80% and a negative predictive value of 67%.

In the whole cohort, 18 neonates had both a high TSB and a high MSB, and in 16 out of 18, the outcome was abnormal. In addition, five out of seven neonates who died, 10 out of 12 neonates with CP, and all five neonates with later epilepsy had either high TSB or both high TSB and high MSB.

### Assessment of seizure burden and outcome in the seizure subgroup (29 neonates)

**Figure 1:**
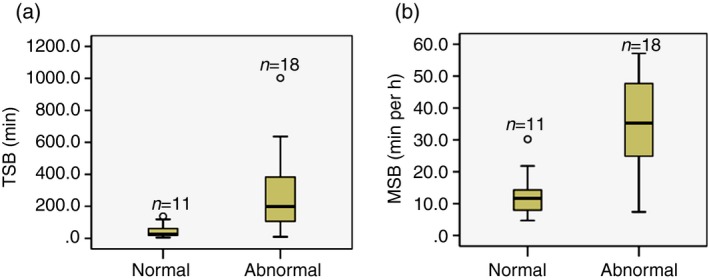
Increased seizure burden in neonates with abnormal neurodevelopmental outcome in the seizure subgroup (*n*=29). (a) Total seizure burden (TSB) in neonates with normal and abnormal outcomes, Mann–Whitney *U* test, *p*<0.001. (b) Maximum seizure burden (MSB) in neonates with normal and abnormal outcomes, Mann–Whitney *U* test, *p*<0.001.

In the subgroup of neonates with seizures, the median TSB and MSB remained significantly higher in those with an abnormal outcome (*p*<0.001 for both; Table 4 and Fig. 1). For every 1‐minute increase of TSB, the odds of the abnormal outcome increased by 2.2% (OR 1.022; 95% CI 1.005–1.039, *p*=0.011) and for every 1‐minute increase of MSB the odds of the abnormal outcome increased by 16% (OR 1.16; 95% CI 1.04–1.30, *p*=0.008). Neonates with abnormal development had significantly longer seizure periods (*p*=0.002), reflecting the increase in seizure burden. The MSB was significantly later after birth in the abnormal outcome group (*p*=0.024) (Table 4).

**Table 4:** Seizure characteristics of neonates with normal and abnormal outcomes in the seizure subgroup (*n*=29)

	Normal outcome (*n*=11)		Abnormal outcome (*n*=18)		*p* a
	Median	IQR	Median	IQR	
TSB (min)	25.4	14.8–78.20	198.7	96.0–390.8	<0.001
MSB (min)	11.7	7.3–15.2	35.3	23.8–48.3	<0.001
Seizure onset (h after birth)	13.2	10.2–15.3	16.7	12.6–20.9	0.173
Seizure period (h)	10.3	0.4–15.6	35.3	18.2–53.0	0.002
MSB (h after birth)	16.9	14.4–22.0	20.4	18.2–32.3	0.024

a From Mann–Whitney *U* test. IQR, interquartile range; TSB, total seizure burden; MSB, maximum seizure burden.

## Discussion

In our cohort of 47 infants with electrographically confirmed moderate or severe HIE, the presence of seizures alone was not associated with abnormal outcome at 24 to 48 months; however, a TSB of more than 40 minutes and an MSB of more than 13 minutes per hour were associated with a significantly higher risk of abnormal development, independent of the application of therapeutic hypothermia or electrographic HIE grade at 24 hours after birth.

In the unadjusted analysis of the subgroup of neonates with seizures (*n*=29), the median TSB and MSB in neonates with abnormal development remained significantly higher.

These results indicate that, in HIE, a high accumulation of seizures plays a more significant role for long‐term developmental outcome than the presence of seizures per se.^17^

The existing physiological evidence from numerous clinical and preclinical studies may help to explain why a high seizure burden increases neurological deficit after HIE. Neonatal seizures have been shown to cause acute local energy depletion, alteration of cerebral blood flow and nutrient supply, and to induce neuronal disintegration.^2^, ^3^ Seizure propagation, and rapid or prolonged accumulation of seizures, results in more significant energy depletion and subsequent apoptosis.^1^

Increased apoptosis and impaired neurogenesis after prolonged neonatal seizures have been reported in animal models of hypoxia.^18^ Recurrent neonatal seizures in rodents were linked to the formation of excessive excitatory circuits in the hippocampus and later epilepsy.^19^ A pathophysiological cascade initiated by prolonged or recurrent seizures may contribute to persistent neuronal inflammation,^1^ which has been proposed as an important mechanism in the pathogenesis of CP.^20^ In our cohort, 10 of the 12 neonates who survived with CP, and all five neonates who subsequently developed epilepsy, had either a high TSB or MSB.

Before the advent of therapeutic hypothermia, studies demonstrated contradictory findings between the presence of seizure and outcome,^6^, ^8^, ^9^ although status epilepticus, defined as seizure burden of more than 20 to 30 minutes per hour, was consistently shown to be associated with unfavourable long‐term and short‐term outcomes.^5^, ^6^, ^21^ The importance of neonatal seizure burden for outcome has been highlighted by many researchers,^2^, ^7^, ^9^, ^22^ but in HIE it has not been fully quantified for long‐term outcome.^7^, ^12^, ^14^ In critically ill children with diseases of mixed aetiologies, including neonates with HIE, MSB of more than 12 minutes per hour was found to be associated with an increase in short‐term neurological decline, independent of the diagnosis or severity of illness.^22^ We have shown that, in HIE, an MSB of more than 13 minutes per hour is associated with poor long‐term outcome, which is conspicuously less than the traditional definition of status epilepticus (seizure burden ≥30min/h)^23^ and slightly less than the threshold of 15 minutes per hour confirmed for aEEG seizures in relation to greater cerebral injury on MRI in a cooled HIE cohort.^12^

Therapeutic hypothermia decreases overall neuronal excitability and seizure propagation, downregulates cerebral metabolism and inflammation, and reduces cerebral necrosis and apoptosis.^1^ However, current protocols for therapeutic hypothermia are more effective in moderate than severe encephalopathy,^1^ and the TSB is significantly reduced in neonates undergoing therapeutic hypothermia with clinically moderate but not severe HIE.^10^ In HIE cohorts with hypothermia, seizures on cEEG were associated with more severe structural brain damage, although 40% of neonates with seizures may have a normal MRI.^13^, ^14^

Our findings suggest that a high seizure burden is associated with an abnormal outcome, irrespective of therapeutic hypothermia. While therapeutic hypothermia reduces seizure burden, the reduction in some neonates may not be sufficient to significantly improve outcome.^1^ Future advances in therapeutic hypothermia protocols may enhance neuroprotective and antiepileptic effects of hypothermia. Clearly, further research is needed to improve AEDs for neonates to help reduce seizure burden in HIE.

The difference in TSB between our neonates treated with therapeutic hypothermia and those without therapeutic hypothermia was dramatic in absolute figures (median 55.8min vs 183.2min), but did not reach statistical significance in the group overall, owing to the high variability in seizure burden across neonates. However, the overall trend for lower seizure burden in our neonates undergoing hypothermia remained, and in our hypothermic subgroup with severe HIE the TSB was significantly lower.

The detrimental effect of a high seizure burden on outcome in our cohort was also independent of the severity of HIE assessed electrographically at 24 hours after birth. Unfortunately, the size of our cohort did not allow for simultaneous control for therapeutic hypothermia and HIE grade.

This is a retrospective study and, as such, the sample size was limited to the number of neonates with moderate and severe HIE collected in the study periods; confidence intervals for the parameters of interest were wide.

Neuroimaging was not available for most of the non‐therapeutic hypothermia cohort; therefore, further evaluation of structural brain injury was not possible. MRI assists in the prediction of the severity and type of neurodevelopmental delay;^17^ however, electrographic grade of HIE has been shown to be as useful as MRI for evaluating the severity of brain injury after perinatal asphyxia.^4^, ^17^

We assessed motor, language, and cognitive function at 24 to 48 months. Motor disability, or general severe disability in HIE, are usually correctly diagnosed by 2 years of age,^24^ while the assessment of cognitive skills and behaviour is more accurate at 6 to 7 years.^25^

Also, children were assessed using two different methods of outcome assessment (Griffiths and Bayley‐III scales), which are not directly comparable. However, both methods provided a validated structured neurodevelopmental assessment.

A large proportion of our neonates received AEDs during their clinical course. A similar number of neonates from our hypothermic and normothermic cohorts were treated with AEDs, but neonates treated with therapeutic hypothermia more frequently received one AED, while neonates not treated therapeutic hypothermia received three or more AEDs. This most probably reflects the increased seizure burden in the non‐therapeutic hypothermia group.^10^ The potential effects of AEDs on seizure burden and EEG background were not analysed in our cohort.

Finally, cEEG monitoring in the non‐therapeutic hypothermia group was less prolonged, and therefore some seizures in this group might have been missed, although their TSB was higher and the length of cEEG in our cohort was sufficient to detect most seizures.^12^, ^13^

Despite limitations, this study expands our knowledge about the relationships between neonatal electrographic seizures and long‐term outcome in HIE, and attempts to control for the severity of injury and the application of therapeutic hypothermia.

## Conclusion

In infants with moderate and severe HIE, a high electrographic seizure burden is significantly associated with abnormal outcome at 24 to 48 months, independent of the severity of encephalopathy or treatment with hypothermia.
